# Associations between family structure and adolescents’ food habits

**DOI:** 10.1017/S1368980020004334

**Published:** 2022-03

**Authors:** Anne-Siri Fismen, Otto Robert Frans Smith, Oddrun Samdal, Arnfinn Helleve, Ellen Haug

**Affiliations:** 1 Department of Health Promotion, Norwegian Institute of Public Health, Bergen 5015, Norway; 2 Department of Health Promotion and Development, University of Bergen, Bergen, Norway; 3 Centre for Evaluation of Public Health Measures, Norwegian Institute of Health, Oslo, Norway; 4 NLA University College, Bergen, Norway

**Keywords:** Family structure, Adolescents, Food habits

## Abstract

**Objective:**

To investigate family structure differences in adolescents’ consumption of fruit, vegetables, sweets and sugar-added soft drinks with adjustments for socio-demographic and socio-economic variables.

**Design:**

Cross-sectional data from the Health Behaviour in School-aged Children survey.

**Setting:**

Norwegian primary and secondary schools.

**Participants:**

Adolescents (*n* 4475) aged 11, 13, 15 and 16 years.

**Results:**

After adjusting for covariates, living in a single-mother family was associated with lower vegetable consumption (OR 0·76, 95 % CI 0·63, 0·91) and higher soft drink consumption (OR 1·29, 95 % CI 1·06, 1·57). Living in a mother and stepfather family was negatively associated with fruit (OR 0·71, 95 % CI 0·54, 0·95) and vegetable (OR 0·72, 95 % CI 0·54, 0·97) consumption. Living in a single-father family was associated with lower sweets consumption (OR 0·48, 95 % CI 0·32, 0·72). No significant interactions were demonstrated between family structure and socio-demographic or socio-economic covariates.

**Conclusions:**

The study suggests that an independent association between family structure and adolescents’ food habits exists.

Diet is one of the important risk factors for overweight and chronic diseases^(1)^. It is therefore of concern that a large proportion of adolescents do not correspond with international recommendations of daily intake of fruit and vegetables accompanied by low intake of sweets and sugar-added soft drinks^(2,3)^. Adolescence is a period when young people gain increased behavioural autonomy regarding their eating habits, and potentially lifelong food preferences and habits are established. However, the wide range of social, cultural, physical and economic environments in which they live influences their diets. Identifying factors and settings associated with adolescents’ food consumption may contribute to an increased understanding of the mechanisms of young people’s eating habits.

The family context has been highlighted as essential when addressing pathways of young peoples’ eating habits^(4–6)^. However, family structures are increasingly diverse in their compositions, and the traditional family consisting of two married parents and their biological children cannot be considered as the sole main family structure. Lately, an increasing part of the youth population lives in a one-parent family or in a reconstructed family consisting of one parent and a step-parent^(7)^, while others live with grandparents or in foster care. Only a limited number of studies have responded to this societal trend by investigating how family structure relates to young people’s eating habits. These studies report a higher intake of fruit and vegetables^(8–10)^ and lower consumption of sugar-added soft drinks^(9)^, potato chips^(11)^ and more regular meal frequency^(9,12–17)^ in adolescents living with both parents. The same pattern is observed for the relationship between BMI and family structure^(10,18)^.

Research on family structure differences draws a complicated picture of family conditions and processes that are associated with health behaviours. Importantly, family structure is associated with a range of adolescent risk behaviours, with those living in nuclear families generally faring best^(13,19–24)^. Although the underlying mechanisms of family structure inequalities are not fully understood, previous studies have suggested that additional differences in time and financial means strongly contribute to explain the general advantages of living in a dual household family^(25,26)^. While single parents may struggle with both time and economy when aiming to facilitate their children with healthy food, dual household families have higher income^(27,28)^ and thereby greater ability to overcome the economic barriers to buy healthy expensive food. Although this perspective draws a parallel to socio-economic determinants^(29)^, studies evaluating possible interaction effects between family structure and socio-economic status (SES) on food habits are lacking. Further, the extent to which step-parent families or if parental gender plays a role in family structure differences has so far not been extensively examined. Previous studies on family structure inequality have almost exclusively defined family structure as simply single- or dual-parent families, and thereby ignoring the potential differences between single-mother and single-father families, and between traditional dual-parent families and reconstituted families that include a step-parent or a parent’s partner. Studies exploring several characteristics of family structures may provide valuable contributions when aiming to understand the importance of contextual determinants of adolescents’ eating habits.

In line with young people in the European Union^(7)^, adolescents in Norway grow up in a variety of family constellations, with an increasing percentage not living together with both parents^(30)^. A fairly common view holds that children and adolescents’ risks of negative outcomes associated with family dissolution are generally small or even non-existent in the Norwegian welfare state, in which family policies and welfare benefits for single parents are well established^(31)^. However, higher BMI, a correlate of unhealthy food patterns, is reported more frequently among Norwegian children with divorced parents compared with children of married/cohabitating parents, or other adult caregivers^(18)^. Further, a recent study showed that living with a single parent or in reconstituted families was unfavourably associated with physical activity, sports participation and screen-based behaviours among Norwegian youth^(32)^. As far as we know, no study has examined the association between family structure differences and adolescents’ dietary habits in a Norwegian sample. Such studies are highly relevant for policymakers and others aiming to improve dietary habits among adolescents across all social groups.

The present study aims to investigate the association between family structure and intake of fruit, vegetables, sweets and sugar-added soft drinks in Norwegian adolescents aged 11, 13, 15 and 16 years, with adjustments for socio-demographic and socio-economic variables.

## Method

### Study design and data collection

The present study reports nationally representative data from the Norwegian part of the international collaborative cross-national Health Behaviour in School-aged Children survey 2014. The overall aim of the Health Behaviour in School-aged Children study is to enhance the understanding of young people’s health behaviours in their social settings. In the current study, school class was the primary sampling unit and a sample of 11 (*n* 1353), 13 (*n* 1030), 15 (*n* 869) and 16 (*n* 1223)-year-old schoolchildren (*n* 4475) participated. The sample was randomly selected using a standard cluster sampling procedure based on a geographical stratified list and sequentially selection from a randomised starting point. The class level response rate was 21 % and the individual student level response rate was 76 %. At a school/class level, a high workload and frequent requests regarding survey participation were reported as the main reasons for non-participation. Absence on the day the survey was conducted was the most frequent cause of non-response at the student level. The Norwegian Western Regional Ethical Committee approved the study and the use of passive consent. A detailed information letter was given both in paper form and electronically to parents or custodians for all participants below the age of 16. Those who did not want their child to participate had to sign and return a form to the teacher. Approval of the child’s participation was assumed if the form was not returned. The students answered the internationally developed, self-administered questionnaire in the classroom (45 min) after receiving standardised instruction from their teacher. Participation was voluntary, and the anonymity, as well as the confidentiality of the participants, was ensured. The questionnaire did not include school/class level variables. More details on the Health Behaviour in School-aged Children study procedures can be found elsewhere^(33)^.

### Measures

*Food habits*, measured as the consumption of fruit, vegetables, sweets and sugar-added soft drinks, were assessed by the item: ‘How many times a week do you eat fruit/vegetables/sweets (e.g. chocolate or candies)/sugar-added soft drinks (e.g. cola or other beverages that contains sugar)?’ ‘Never’, ‘Less than once a week’, ‘Once a week’, ‘Two to four times a week’, ‘Five to six times a week’, ‘Once a day’, ‘More than once a day’. The measurements have been recognised as a valid instrument in epidemiological studies ranking adolescents according to their usual food intake^(34)^.

*Family structure* was assessed with the item ‘Please answer this first question for the home where you live all or most of the time and tick the people who live there’. The response categories were ‘Mother’, ‘father’, ‘stepmother (or father’s partner)’, ‘stepfather (or mother’s partner)’ and ‘other (e.g., living with grandparents and adults other than their parents such as foster parents or care homes)’. These were categorised into ‘both parents’, ‘single mother’, ‘single father’, ‘mother and stepfather’, ‘father and stepmother’ and ‘other’.

*SES* was measured by a summary score of The Family Affluence Scale III (FAS-III)^(35)^. The Family Affluence Scale III contains six items: (1) Does your family own a car, van or truck? (responses: no, one, two or more); (2) Do you have your own bedroom for yourself? (no, yes); (3) How many times did you and your family travel out of Norway for a holiday/vacation last year? (not at all, once, twice, more than twice); (4) How many computers do your family own? (none, one, two, more than two); (5) Does your family have a dishwasher at home? (no, yes) and (6) How many bathrooms (rooms with a bath/shower or both) are in your home? (none, one, two, more than two). A ridit transformation (conversion to cumulative probabilities) by age and sex was carried out on the sum score of the Family Affluence Scale III items. The ridit scores were then categorised into three groups with varying levels of relative material affluence: low (lowest 20th percentile), medium (between 20th and 80th percentile) and high (highest 20 %).

*BMI* (kg/m^2^) was calculated using self-reported weight and height assessed by two items: ‘How much do you weigh without clothes?’ (in kg) and ‘How tall are you without shoes?’ (in cm). The BMI scores were recoded into standardised *z*-scores as recommended by the International Obesity Task Force^(36)^.

*Siblings* were assessed by two items referring to the household the participants lived all or most of the time: ‘Please indicate how many brothers and sisters live here (including half, step or foster brothers and sisters)’ (How many brothers?; How many sisters?).

### Statistics

Ordered logistic regression models were used to examine the associations between food habits (modelled as ordinal outcome variables) and the dummy-coded family structure variable with ‘both parents’ as the reference category. Previous research has demonstrated the associations between adolescents’ food habits and age, gender, BMI, number of siblings and SES^(4–6,31)^. Against this background, these variables were included as covariates in the adjusted analyses. In the adjusted model, interactions between family structure and the included covariates were tested on a one by one basis. Likelihood ratio tests were used to determine whether the included interaction effect was significant at the *P* = 0·05 level.

Ordered logistic regression assumes that the coefficients that describe the association between the highest *v*. all lower categories of the response variable are the same as those that describe the association between the second-highest and all lower categories, known as the proportional odds assumption. To test this assumption, adjusted partial proportional odds models (gologit2 in Stata) were run for each outcome^(37)^. An overall Wald test significant at the *P* < 0 05 level was interpreted as a violation of the proportional odds assumption. In this case, the proportional odds constraints were relaxed for those variables that violated the assumption. All statistical procedures were performed in Stata version 14.0.

## Results

The characteristics of the sample are presented in Table 1. About one quarter reported living in a single-parent family or a step-parent family. As shown in Table 2, the majority did not report daily intake of fruit and vegetables. Around 50 % reported eating sweets and sugar-added soft drinks more than once a week.

**Table 1:** Characteristics of the study population (*n* 4475)

Variable	Estimate	
	%	*n*
Female	52·8	2381
Age categories		
11 years	30·0	1353
13 years	22·8	1030
15 years	19·3	869
16 years	27·1	1223
BMI *Z*-score		
Mean	19·7	
sd	3·5	
Family structure		
Both parents	74·1	2962
Single mother	12·5	498
Single father	3·3	130
Mother and stepfather	4·8	192
Father and stepmother	1·1	51
Other family structures*	4·1	161
SES†		
Low SES	18·3	783
Middle SES	65·5	2809
High SES	16·2	696
Have at least one sibling	90·9	3652

SES, socio-economic status.

* Other family structures include grandparents and adults other than their parents such as foster parents or care homes.

† SES refers to ridit transformation of summary score on Family Affluence Scale.

**Table 2:** Frequency of food intake by age groups (*n* 4272)

	11 years		13 years		15 years		16 years	
	%	*n*	%	*n*	%	*n*	%	*n*
Fruit consumption								
Never	1·4	17	2·2	22	3·2	26	2·1	25
Less than once a week	5·0	63	6·9	69	6·1	49	6·8	81
Once a week	6·9	87	8·5	85	10·7	86	12·5	148
2–4 d a week	21·5	270	25·3	253	26·0	209	31·7	375
5–6 d a week	20·7	261	20·4	204	17·7	142	17·7	209
Once daily	19·9	250	17·5	175	17·0	137	12·4	147
More than once daily	24·6	310	19·3	193	19·3	155	16·7	198
Vegetable consumption								
Never	3·3	41	3·6	36	3·2	36	3·1	37
Less than once a week	5·3	67	5·2	52	5·2	52	5·0	59
Once a week	6·7	84	9·9	99	8·7	99	8·5	100
2–4 d a week	22·5	282	23·8	238	26·2	238	28·4	336
5–6 d a week	21·4	269	22·8	228	24·3	228	26·4	312
Once daily	23·3	293	22·0	220	20·6	220	16·2	192
More than once daily	17·5	220	12·7	127	11·7	127	12·4	147
Sweets consumption								
Never	5·7	72	2·8	28	2·7	22	4·2	50
Less than once a week	15·0	189	8·5	85	10·7	86	12·8	151
Once a week	46·4	583	36·7	367	26·4	212	29·9	354
2–4 d a week	27·0	339	40·4	404	43·5	350	39·7	470
5–6 d a week	2·5	32	6·1	61	10·0	80	7·4	88
Once daily	1·6	20	2·8	28	3·6	29	4·1	49
More than once daily	1·7	21	2·7	27	3·1	25	1·8	21
Sugar-added soft drink consumption								
Never	8·4	105	5·5	55	7·0	56	10·4	123
Less than once a week	21·8	274	15·5	155	14·1	113	19·4	230
Once a week	37·3	468	29·9	299	26·6	214	22·3	264
2–4 d a week	25·2	317	37·1	371	34·0	273	30·6	362
5–6 d a week	3·4	43	6·6	66	10·0	80	9·6	114
Once daily	1·9	24	2·2	22	3·9	31	3·2	38
More than once daily	2·0	25	3·2	32	4·6	37	4·4	52

### Associations between family structure and adolescents’ food habits

Crude associations were demonstrated between single-mother families and consumption of fruit, vegetables and sugar-added soft drinks, between single-father families and consumption of fruit and sweets and between mother and stepfather families and consumption of fruit and vegetables. Except for the association between single-father families and fruit consumption and also the associations between single-mother families and fruit consumption, the associations remained significant in the adjusted models (Table 3). No significant interactions between family structure and the included covariates were found (all likelihood ratio tests *P* > 0·05). More details from the adjusted main effects analyses are provided below.

**Table 3:** Crude and adjusted model for associations between family structure and adolescents’ food habits

	Fruit consumption (*n* 3974)		Vegetable consumption (*n* 3972)		Sweets consumption (*n* 3972)		Soft drink consumption (*n* 3972)	
	OR	95 % CI	OR	95 % CI	OR	95 % CI	OR	95 % CI
Crude model								
Family structure								
Single mother	0·79	0·65, 0·96	0·75	0·63, 0·89	1·14	0·96, 1·34	1·21	1·01, 1·45
Single father	0·62	0·46, 0·85	0·76	0·56, 1·04	0·68	0·47, 0·98	1·02	0·67, 1·55
Mother and stepfather	0·70	0·54, 0·91	0·74	0·57, 0·97	1·13	0·85, 1·49	1·15	0·87, 1·52
Father and stepmother	0·60	0·33, 1·09	0·74	0·44, 1·24	0·59	0·35, 1·02	0·76	0·48, 1·21
Other family structure	0·88	0·68, 1·15	0·89	0·69, 1·15	1·04	0·78, 1·37	1·16	0·90, 1·50
Adjusted model								
Female	1·79	1·56, 2·05	1·43	1·26, 1·61	0·91	0·80, 1·04	0·48	0·42, 1·21
Age categories								
13 years	0·71	0·58, 0·85	0·74	0·61, 0·89	2·12	1·71, 2·64	1·70	1·38, 2·10
15 years	0·67	0·55, 0·82	0·70	0·57, 0·86	3·10	2·46, 3·90	2·05	1·65, 2·56
16 years	0·52	0·98, 1·02	0·65	0·53, 0·79	2·47	1·94, 3·14	1·69	1·34, 2·15
BMI	1·00	0·98, 1·02	1·00	0·98, 1·02	0·96	0·93, 0·98	0·98	0·96, 1·00
Family structure*								
Single mother	0·85	0·68, 1·06	0·76	0·63, 0·91	1·11	0·93, 1·32	1·29	1·06, 1·57
Single father	0·82	0·57, 1·19	1·06	0·75, 1·50	0·48	0·32, 0·72	0·72	0·47, 1·12
Mother and stepfather	0·71	0·54, 0·95	0·72	0·54, 0·97	1·06	0·77, 1·46	1·23	0·89, 1·69
Father and stepmother	0·79	0·39, 1·63	0·92	0·81, 1·66	0·56	0·29, 1·11	0·81	0·50, 1·32
Other family* structure	0·75	0·56, 1·02	0·79	0·57, 1·07	0·93	0·69, 1·26	1·15	0·87, 1·53
SES**								
Middle SES	1·17	1·00, 1·37	1·18	0·99, 1·40	1·06	0·89, 1·27	1·17	0·98, 1·40
High SES	1·67	1·32, 2·10	1·68	1·34, 2·11	1·05	0·82, 1·34	1·07	0·84, 1·37
At least one sibling	0·97	0·78, 1·21	1·00	0·81, 1·23	0·99	0·81, 1·21	0·89	0·73, 1·09

SES, socio-economic status.

The following reference categories were used: gender: male, age: 11 year olds, family structure: living with both parents, SES: low SES.

* Other family structures include grandparents and adults other than their parents such as foster parents or care homes.

** SES refers to ridit transformation of summary score on Family Affluence Scale.

#### Single parent family

The adjusted analyses showed that adolescents living in single-mother families reported a lower intake of vegetables (OR 0·76, 95 % CI 0·63, 0·91) and higher intake of sugar-added soft drinks (OR 1·29, 95 % CI 1·06, 1·57) compared with their counterparts living with both parents. No significant associations were found between single-mother families and consumption of fruits and sweets. Adolescents living in single-father families reported a lower intake of sweets (OR 0·48, 95 % CI 0·32, 0·72), compared with their counterparts living with both parents. Living in a single-father family was not associated with the intake of fruit, vegetables or sugar-added soft drinks.

#### Step-parent family

The adjusted analyses showed that adolescents living in mother and stepfather families reported lower intake of fruits (OR 0·71, 95 % CI 0·54, 0·95) and vegetables (OR 0·72, 95 % CI 0·54, 0·97), compared with their counterparts living with both parents. No significant associations were found between mother and stepfather families and the consumption of sweets and sugar-added soft drinks. Living in a father and stepmother family was not significantly associated with adolescents’ food habits.

#### Other parent family

No significant associations were found between the category ‘other parent family’ and adolescents’ food habits.

#### Age and gender differences

As shown in Table 3, female gender was associated with higher consumption of fruit and vegetables. Higher age was associated with lower intake of fruit and vegetables and higher intake of sweets and sugar-added soft drinks.

Results from the partial proportional odds models indicated that the adjusted models did not violate the proportional odds assumption for the outcomes of fruit, vegetables and sweets consumption. However, for soft drink consumption, the overall Wald test was significant (*χ* ^2^ (40) = 56·16, *P* = 0·046). For family structure, only the ‘single mother’ category violated the proportional odds assumption in relation to soft drink consumption. The direction of the association was the same for all outcome levels, but the OR varied between 1·14 and 2·08. As compared with both parent families, single-mother families were particularly more likely to be in the ‘once daily or more’ category (OR = 2·08, 95 % CI 1·48, 2·95) and the ‘more than once daily’ category (OR = 1·92, 95 % CI 1·16, 3·16).

## Discussion

The present study adds to the research on family structure differences by demonstrating the associations between family type and adolescents’ food habits using Norwegian nationally representative data. After adjusting for age, gender, BMI, SES and having siblings, the results showed that living in a single-mother family was associated with lower vegetable consumption and higher soft drink consumption. Living in a mother and stepfather family was negatively associated with fruit and vegetable consumption. Living in single-father families was favourably associated with lower sweet consumption.

Less favourable food habits in single-parent families and step-parent families are shown in other studies^(8–10)^. Although explaining pathways of family structure inequalities is beyond the scope of this paper, it is worth considering why national dietary recommendations may be more easily achieved in some family types than others. One possible explanation for the less favourable food habits among adolescents living in single-mother families could be that family structure is a proxy for SES. A social gradient in single parenthood is reported in Norway as well as other European countries; single mothers are shown to be lower educated and more likely to face material deprivation than do mothers in dual-headed households^(29,38,39)^. Lower parental SES is associated with lower vegetable consumption and higher soft drink consumption^(40)^. Vegetables are expensive food items in Norway, and the finding of lower vegetable consumption among adolescents living in single-mother families might reflect that cost is a barrier of particular importance to single mothers. However, adjustments for SES were included in the present analysis. Lower OR for vegetable consumption was observed in low *v*. high SES groups, but no significant interaction effects were identified between family structure and SES. The present findings are in line with a systematic review of family structure differences^(41)^ in which it was concluded that family structure differences persist after adjusting for material wealth. This suggests that family structure differences are driven by other underlying mechanisms than the one represented by the material dimension of SES.

Another explanation is that time and routines for family meals differ across the different family types, and that healthy food habits may be more easily established in families where there are two parents present. While fruits are ready to eat, vegetables do often require time and preparation before they can be consumed. In single-parent families, where a larger number of responsibilities are placed on one parent, routines of food preparation may be less achievable. Vegetables are usually included in Norwegian dinner and evening meals and the findings of lower vegetable consumption in single-mother families might indicate fewer prepared family meals, replaced by faster alternatives containing fewer vegetables. However, the number of parents in the family does not in itself explain the observed difference in vegetable consumption, as the current study presents the lowest OR of regular fruit and vegetable consumption among adolescents living in mother and stepfather families. This might seem surprising as stepfathers contribute with both time and economic resources and may thereby participate in cooking procedures and improve the family’s ability to buy healthy food^(42)^. On the other hand, it has been suggested that step-parents may underinvest in non-biological children, because they may be providing resources to their prior biological children in other households or because they are less committed to non-biological children^(43)^. Further, reconstituted households are more likely to have one or more strained parent–child relationships^(44)^, which is important as family cohesion is positively associated with healthy family diets^(15)^. Only mother and stepfather, and not father and stepmother, were associated with lower fruit and vegetable consumption. As only 1·1 % (*n* 51) reported living in a father and stepmother family, this finding should be interpreted with caution, as the low number might have biased our results.

The current study suggests that adolescents living in single-father families are less likely to consume sweets on daily basis than do adolescents living with both parents. The findings suggest the role of the father to be more important than previous studies that have reported that fathers spend less time on cooking and are less concerned about their children’s diet^(45,46)^. Traditional gender roles have merged and based on the present results, one can argue that it is time for reconsideration of fathers’ ability to facilitate and promote healthy food habits among adolescents. Another possible explanation is that single fathers are a selected group of men and that the low engagement in children’s food habits reported in fathers^(46)^ cannot be generalised to single fathers. The lower intake of sweets among adolescents living in single-father families may be further explained by role modelling. Fathers’ dietary intake is positively associated with their children’s food habits^(47)^, and men are generally showing lower intake of sweets and sugary food than do women^(48)^. Gender differences in food habits were seen also in the current study population. In line with previous research, higher fruit and vegetable consumption was observed among girls than boys^(49)^.

### Implications

The present results support previous research in which family structure differences were associated with young people’s eating habits^(8–10)^. However, while previous studies reported advantages of living in dual-parent *v*. single-parent families, the current study explored the role of family composition in greater details. The present findings underscore that the number of caregivers does not alone explain dietary differences. Family cohesion might be an important mediator of family structure differences, and psychosocial conditions may contribute to explain the family structure differences observed. Our findings of lower vegetable consumption among children living in single-mother families contrast a previous US study in which increased vegetable consumption was reported in families with non-resident father involvement^(9)^. This might indicate that the importance of family structure differences varies across countries, a perspective that should be investigated in future studies. Also, possible associations between family structure and parental food habits, time used for cooking and frequencies of meals eaten in and out of the home, takeaway meals, etc. should be investigated. Still, the results highlight the relevance of incorporating the importance of family context in initiatives targeting food habits among adolescents.

Health professionals and policymakers should keep in mind that young people’s family context can be complex and have the potential to affect young people’s diets. The present study suggests that the Norwegian welfare policy does not eliminate family structure differences and that the role of family cohesion should be addressed in public nutrition initiatives. Further, as the proportion of children and adolescents living in single-parent or step-parent families continues to grow in Norway as well as in other countries, it is important to monitor food habits by family structure, alongside overall population trends. Attention should be devoted to a trend study in which family structure inequalities in eating habits were shown to increase^(46)^. Finally, the present findings should be viewed from a broader public health perspective, as unfavourable diets add to a range of negative health behaviours, for example, physical inactivity, smoking and substance use, identified among adolescents living in single-parent or step-parent families^(13,19–24)^.

### Limitations

There is a risk that students absent on the day the survey was conducted are characterised by special food habits or family structures. The presented associations might hence be underestimated. We were not able to differentiate between those living with a single parent with no involvement from the other parent and those living most of the time with, for example, mother and part-time with father. Parental age was not measured in the current study but could potentially have influenced the results. Also, peer influence could potentially have biased the results. The present findings should thus be interpreted in light of this limitation. Further, the findings of no significant interactions between family structure and SES might be relatively more applicable for egalitarian welfare states such as Norway compared with other countries where socio-economic factors might represent greater barriers to single-parent families. Further, the presented results of the interaction analysis could be different if Family Affluence Scale III was replaced with another SES indicator, for example, parental education, parental occupation or family income.

## Conclusion

As far as we know, the current study is the first to examine the association between family structure and adolescents’ food habits in a Norwegian sample. The study provides insight on family structure differences, suggesting that adolescents living in single-mother families and mother and stepfather families have a lower likelihood of experiencing the benefits of healthier food habits compared with adolescents living with both parents. The study also underlines that the traditional or nuclear family structure cannot be considered as the sole or indeed family composition when exploring adolescents’ food habits.
